# Prevalence of chronic pain among U.S. children with neurodevelopmental disorders

**DOI:** 10.1097/PR9.0000000000001495

**Published:** 2026-09-01

**Authors:** Hannah Chou, Laila Amina Chaudhry, Jennifer A. Rabbitts, Cornelius Botha Groenewald

**Affiliations:** a Rush University, Chicago, IL, USA; b Department of Anesthesiology, Perioperative, and Pain Medicine, Stanford University School of Medicine, Stanford, CA, USA

**Keywords:** Chronic pain, Neurodevelopmental disorders, Pediatric pain, Population-based study

## Abstract

**Introduction:**

Neurodevelopmental disorders (NDDs) affect up to 15% of children in the United States and are associated with co-occurring health challenges such as chronic pain, which can negatively affect development and quality of life. Increased risk of chronic pain in children with NDDs remains poorly understood; this study aimed to estimate chronic pain prevalence among children with NDDs and explore whether this association differs by sex.

**Methods:**

We conducted a cross-sectional analysis of children participating in the 2022 to 2023 National Survey of Children's Health (age 3–17 years, n = 84,117). Neurodevelopmental disorders included attention deficit and hyperactivity disorder, autism, intellectual disability, speech disorder, Tourette syndrome, learning disability, and developmental delay. Chronic pain was defined as frequent or persistent pain in the past 12 months. Weighted multivariable logistic regression estimated associations between NDDs and chronic pain, adjusting for sociodemographic and health covariates.

**Results:**

Overall, 18.7% (95% confidence interval: 18.2%–19.2%) of children had an NDD. Chronic pain prevalence was higher among children with NDDs than among those without (10.3% vs 5.2%, *P* < 0.001). After adjustment, children with any NDD (aOR 1.9 [1.7–2.2]) had increased odds of chronic pain. Tourette syndrome showed the greatest odds (aOR = 3.2 [1.7–6.2]). Sex did not alter the association between NDDs and chronic pain (*P* = 0.79).

**Conclusion:**

Neurodevelopmental disorders affect 1 in 6 U.S. children and are associated with almost double the odds of chronic pain. This increased odds emphasizes the need for improved chronic pain recognition and management among children with NDDs. Future research should examine chronic pain in youth with NDDs, to improve assessment, management, and understanding of pain mechanisms.

## Abstract 2

Children with neurodevelopmental disorders showed nearly double the odds of chronic pain, indicating needs for better pain recognition and tailored pain management in pediatric practice.

## 1. Introduction

Neurodevelopmental disorders (NDDs) are increasingly recognized as a major source of morbidity in childhood, with epidemiological estimates suggesting that up to 15% of children in the United States are diagnosed with at least one NDD.^20^ The public health burden of these conditions is substantial and has been increasing over the past 2 decades.^61^ Despite this, children with NDDs remain an understudied population in pediatric health research, particularly in regard to co-occurring chronic health conditions.

One such condition, chronic pain, defined as persistent or recurring pain for more than 3 months, is associated with significant lifelong consequences for development, daily functioning, and quality of life.^12,52^ For children with NDDs, chronic pain may act as a compounding disability, further impeding developmental milestones and reducing participation in educational and social environments.^44^ When left unaddressed, this elevates the risk for secondary mental health challenges, such as anxiety and depression.^29^

Recent evidence from a survey study using 2016 to 2017 National Survey of Children's Health data indicated a higher prevalence of pain among specific NDD populations such as autism spectrum disorder.^58^ In addition, literature examining youth with brain-based developmental disabilities has suggested an increased burden of acute and chronic pain compared with peers without developmental disabilities.^43^ However, the experience of chronic pain among the broader population of pediatric NDD has not been well described. To date, most research on pediatric chronic pain has focused on neurotypical populations, and investigations into NDDs and chronic pain have focused on single NDDs in isolation, limiting generalizability of findings.^12,38^ This is an important gap in knowledge, as prior research has frequently lacked a consistent or inclusive operational definition of NDDs, making it difficult to compare findings across studies.^46^ Moreover, the high rate of comorbidity among different NDDs may compound the negative health outcomes for this population.^8^ Better understanding of pain prevalence across the NDD spectrum may help clinicians identify children at increased odds for chronic pain and guide more tailored screening and management strategies for children whose pain may otherwise be underrecognized.

Biological sex may influence the relationship between NDDs and chronic pain. Sex differences in pediatric chronic pain are well established. Girls are more likely to report chronic pain and exhibit higher pain sensitivity,^31^ but few studies have examined whether this pattern holds among children with NDDs. Conversely, boys are more frequently diagnosed with NDDs, although this may be influenced by underdiagnosis in girls, who may present with different symptoms or engage in camouflaging behaviors.^24^ Given these well-established sex-dependent patterns, it is critical to examine the influence of sex in the association between NDDs and chronic pain.

The primary objective of this study was to estimate the prevalence of chronic pain in children with NDDs compared with those without NDDs, while accounting for sociodemographic and health factors. Prior studies in selected NDD populations suggest an elevated pain burden,^58^ but the extent to which this pattern applies across the broader pediatric NDD spectrum remains unclear. We hypothesized that children with NDDs would have a higher prevalence of chronic pain than children without NDDs. Our secondary objective was to explore whether the association between NDDs and chronic pain differed by biological sex.

## 2. Methods

This was a cross-sectional analysis using publicly available data from the 2022 and 2023 National Survey of Children's Health (NSCH), a population-based survey conducted annually by the U.S. Census Bureau and sponsored by the Health Resources and Services Administration's Maternal and Child Health Bureau.^55^ The NSCH provides a nationally representative sample of U.S. children age 0 to 17 years and includes parent- or caregiver-reported information on child health, healthcare access, and family and community contexts. National Survey of Children's Health is primarily designed to provide national estimates of physical and mental health of children in the United States. The response rate was 39.1% in 2022 and 35.8% in 2023. Despite these low response rates, nonresponse bias analysis indicated that weighting and adjustment procedures substantially reduced differences between respondents and the target population, suggesting no meaningful nonresponse bias in the study estimates.^55^ Surveys were administered online and by mail using probability-based sampling techniques. To ensure broad accessibility, the NSCH instruments were administered in both English and Spanish. All analyses accounted for the complex sampling design, including weighting, clustering, and stratification. Due to this study being a secondary analysis of deidentified data, it was deemed exempt from review by the Stanford University Institutional Review Board.

### 2.1. Identifying neurodevelopmental disorders

The primary exposure was the presence of any neurodevelopmental disorder, defined according to the diagnostic categories grouped under “neurodevelopmental disorders” in the Diagnostic and Statistical Manual of Mental Disorders, Fifth Edition (DSM-5).^2^ These include intellectual disabilities, communication disorders, autism spectrum disorder (ASD), attention deficit hyperactivity disorder (ADHD), specific learning disorder, and motor disorders. Applying this definition to the NSCH data, children were considered to have an NDD if their parent or caregiver reported that they currently had one or more of the following conditions: ASD, attention deficit disorder/attention deficit hyperactivity disorder (ADD/ADHD), intellectual disability, speech disorder, Tourette syndrome, learning disability, or developmental delay by responding yes to both questions: (1) “Has a doctor or other healthcare provider EVER told you that this child has [the condition],” and (2) “Does this child currently have the condition?” We intentionally used this standardized approach of DSM-5 classification because these diagnoses share neurocognitive and behavioral features that follow similar care pathways^40^—neurological diseases like cerebral palsy and epilepsy represent structural and neurological conditions outside this DSM-5 NDD class—thereby addressing inconsistencies in prior literature, in which terms like “developmental disorders” were often variably or ambiguously applied, leading to limited generalizability and cross-study comparisons.^46^

### 2.2. Participant demographics

The cohort for our study included children age 3 to 17 years who participated in the 2022 and 2023 NSCH. Children age 0 to 2 years were excluded because the neurodevelopmental disorder measures used in this study are only available for children age 3 to 17 years in the NSCH. We excluded participants with missing data on any of the variables used in multivariable analyses (n = 7,059). Data were available for the following sociodemographic variables: age, sex (male vs female), self-reported race/ethnicity (White, non-Hispanic, Black, non-Hispanic, Hispanic, Asian, non-Hispanic, and other or multiracial non-Hispanic), household income (0%–99% federal poverty level [FPL], 100%–199% FPL, 200%–399% FPL, 400% or greater FPL), insurance type (private, public only, uninsured), and primary language spoken at home (English vs non-English), and U.S. region (Northeast, Midwest, South, West). We also controlled for parent-reported overall child health (rated by parent as excellent/very good, or good/fair/poor).

### 2.3. Chronic pain

Parents were asked, “During the past 12 months, did this child have frequent or chronic difficulty with repeated or chronic physical pain, including headaches or other back or body pain?” The presence of chronic pain was determined by a “yes” response to this item.^28^

### 2.4. Statistical analysis

Analyses were performed using Stata version v18.0 (StataCorp, College Station, TX). α was set at 0.05. Stata's survey design package was used to adjust for the complex sample design of NSCH by using sampling weights, regional stratification, and primary sampling units.

Descriptive statistics for sociodemographic and clinical characteristics were reported as weighted percentages with 95% confidence intervals (Table 1).

**Table 1:** Overall sample characteristics.

	Weighted %	95% CI
Age categories		
3–5 y	18.7	18.3, 19.2
6–11 y	39.5	38.8, 40.1
12–17 y	41.8	41.1, 42.5
Sex of child		
Male	51.2	50.5, 51.8
Female	48.8	48.2, 49.5
Race and ethnicity		
White, non-Hispanic	48.5	47.9, 49.2
Black, non-Hispanic	12.3	11.8, 12.9
Hispanic	26.6	25.9, 27.3
Asian, non-Hispanic	4.9	4.7, 5.2
Other/multiracial, non-Hispanic	7.6	7.3, 7.9
Percent of poverty level		
0%–99% of poverty level	17.5	16.9, 18.1
100%–199% of poverty level	19.3	18.7, 19.9
200%–399% of poverty level	29.3	28.7, 29.9
400% or more of poverty level	33.9	33.3, 34.5
Primary language		
English	85.3	84.6, 85.9
Non-English	14.7	14.1, 15.4
U.S. census region		
Northeast	15.7	15.3, 16.2
Midwest	21.2	20.8, 21.7
South	39.1	38.4, 39.8
West	23.9	23.4, 24.4
Insurance type		
Private	64.5	63.8, 65.2
Public only	29.1	28.4, 29.7
Uninsured	6.4	6.0, 6.9
General health		
Excellent or good	98.5	98.4, 98.7
Fair or poor	1.5	1.3, 1.6
Any neurodevelopmental disorder	18.7	18.2, 19.2
ADD/ADHD	10.6	10.2, 11.0
Autism	3.9	3.6, 4.2
Developmental delay	6.0	5.6, 6.3
Intellectual disability	1.3	1.1, 1.4
Learning disability	7.8	7.4, 8.2
Speech disorder	6.6	6.3, 6.9
Tourette syndrome	0.2	0.2, 0.3
Chronic pain	6.1	5.8, 6.5

ADHD, attention deficit and hyperactivity disorder; CI, confidence interval.

**Figure 1.**
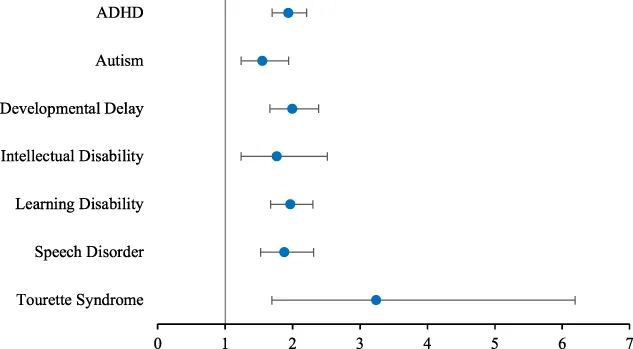
Adjusted odds ratios and 95% confidence intervals for chronic pain among children with specific neurodevelopmental disorder, estimated using survey-weighted multivariable logistic regression models. Models were adjusted for age, sex, race/ethnicity, insurance status, primary language, U.S. census region, and general health status. ADHD, attention deficit and hyperactivity disorder.

To address our primary objective, we first conducted Rao-Scott adjusted χ^2^ tests to compare prevalence rates of chronic pain among children with NDDs to children without NDDs. We also estimated the prevalence of chronic pain in individual NDD disorders, including ADD/ADHD, autism, developmental delay, intellectual disability, learning disability, speech disorder, and Tourette syndrome (Table 2). We next conducted survey-weighted multivariable logistic regression to estimate the association between overall neurodevelopmental disorder status and chronic pain, controlling for age, sex, race/ethnicity, insurance status, primary language, U.S. census region, and general health status (Table 3). We then calculated the association between each individual NDD and chronic pain using survey-weighted multivariable logistic regression. The adjusted odds ratios (aOR) with 95% confidence intervals were displayed in a forest plot (Fig. 1).

**Table 2:** Prevalence of chronic pain among children with and without neurodevelopmental disorders in the United States.

	Chronic pain rates				*P*
	Has disorder		Does not have disorder		
	Weighted %	95% CI	Weighted %	95% CI	
Any neurodevelopmental disorder	10.3	9.5, 11.2	5.2	4.8, 5.5	<0.0001
ADD/ADHD	11.8	10.7, 12.9	5.5	5.1, 5.8	<0.0001
Autism	9.8	8.2, 11.8	6.0	5.7, 6.3	<0.0001
Developmental delay	11.9	10.4, 13.6	5.8	5.4, 6.1	<0.0001
Intellectual disability	16.6	12.8, 21.2	6.0	5.7, 6.3	<0.0001
Learning disability	13.5	12.0, 15.1	5.5	5.2, 5.9	<0.0001
Speech disorder	9.2	7.9, 10.8	5.9	5.6, 6.3	<0.0001
Tourette syndrome	17.7	10.6, 28.1	6.1	5.8, 6.4	<0.0001

Source: NSCH 2022 to 2023.

ADD/ADHD, attention deficit disorder/attention deficit and hyperactivity disorder; CI, confidence interval.

**Table 3:** Multivariate logistic regression analysis demonstrating the association between neurodevelopmental disorder and chronic pain among children and adolescents.

Characteristic	aOR	95% CI	*P*
NDD			
No	Ref		
Yes	1.9	1.7, 2.2	<0.0001
Age category			
3–5 y	Ref		
6–11 y	3.6	2.9, 4.5	<0.0001
12–17 y	8.1	6.5, 10	<0.0001
Sex			
Male	Ref		
Female	1.8	1.6, 2	<0.0001
Race and ethnicity			
White, non-Hispanic	Ref		
Black, non-Hispanic	0.9	0.7, 1.1	0.235
Hispanic	0.9	0.8, 1	0.172
Other/multiracial, non-Hispanic	0.8	0.7, 0.9	0.002
General health			
Excellent or good	Ref		
Fair or poor	7.7	5.9, 10	<0.0001
Poverty level			
0%–99% of poverty level	Ref		
100%–199% of poverty level	1.0	0.8, 1.3	0.819
200%–399% of poverty level	0.9	0.7, 1.1	0.165
400% or more of poverty level	0.6	0.5, 0.7	<0.0001
Insurance			
Private	Ref		
Public only	1.1	1, 1.3	0.09
Uninsured	0.9	0.7, 1.3	0.697
Primary language at home			
English	Ref		
Non-English	1.0	0.8, 1.2	0.9
Geographical region			
Northeast	Ref		
Midwest	1.0	0.9, 1.2	0.73
South	0.9	0.8, 1.1	0.299
West	1.2	1, 1.4	0.03

CI, confidence interval; NDD, neurodevelopmental disorder.

**Figure 2.**
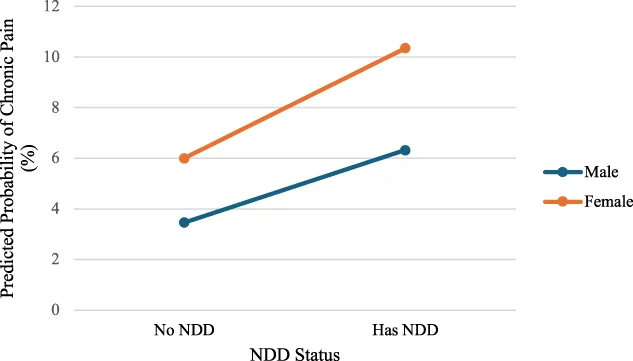
Predicted probability of chronic pain by NDD status and sex, estimated from adjusted survey-weighted multivariable logistic regression models. NDD, neurodevelopmental disorder.

To address our secondary objective, we used a survey-weighted logistic regression model adjusted for sociodemographic covariates with an interaction term between NDD status and biological sex (male vs female). The predicted probabilities of chronic pain for males and females with and without an NDD were displayed in an interaction plot (Fig. 2).

## 3. Results

### 3.1. Sample sociodemographics

Table 1 displays participant sample characteristics. Participants varied across the pediatric age range with 18.7% of participants being 3 to 5 years, 39.5% being 6 to 11 years, and 41.8% being 12 to 17 years. Approximately half were male (51.2%) and half were female (48.8%). The majority were White (48.5%), followed by Hispanic (26.6%), and 85.3% were primarily English speakers. Most participants fell into an income bracket at or above 200% of the poverty level (63.2%), whereas smaller percentages were from 100% to 199% of the poverty level (19.3%) or below the poverty level (17.5%). Participants were representative of the U.S. geographic distribution, with most coming from the South (39.1%), 23.9% from the West, 21.2% from the Midwest, and 15.7% from the Northeast. Most participants (64.5%) had private health insurance, whereas 29.1% only had public health insurance, and 6.4% were uninsured.

### 3.2. Health characteristics

Overall, 98.5% of participants were reported to be in excellent or good general health, while 1.5% were reported to be in fair or poor health. Chronic pain was reported in 6.1% of participants and 18.7% of participants were diagnosed with a neurodevelopmental disorder. Across the full sample, 10.6% were diagnosed with ADD/ADHD, 3.9% with autism, 6% with a developmental delay, 1.3% with an intellectual disability, 7.8% with a learning disability, 6.6% with a speech disorder, and 0.2% with Tourette syndrome.

### 3.3. Prevalence of chronic pain in children with neurodevelopmental disorders

The prevalence of chronic pain was significantly higher among children with an NDD compared with those without (Table 2). Among children with an NDD, the prevalence of chronic pain was 10.3%, compared with 5.2% in those without an NDD (*P* < 0.001). The prevalence of chronic pain varied by type of NDD. The highest prevalence of chronic pain was observed in children with Tourette syndrome (17.7%), followed by intellectual disability (16.6%), learning disability (13.5%), developmental delay (11.9%), ADHD (11.8%), autism (9.8%), and speech disorder (9.2%). Notably, each NDD was associated with higher chronic pain prevalence compared with children without NDDs (*P* < 0.001).

After controlling for demographic variables (age, sex, race/ethnicity, primary language, U.S. census region, health insurance), and health status, individuals with NDD had significantly higher odds of having chronic pain than their non-NDD counterparts (aOR = 1.9, 95% confidence interval [CI] [1.7–2.2], *P* < 0.001; Table 3). Older age, female sex, and fair or poor health were also associated with increased odds of chronic pain, whereas higher household income showed a protective association. Using these adjusted models, odds ratios for individual NDDs were also calculated and are visualized in Figure 1: ADHD (aOR = 1.9, 95% CI [1.7, 2.21]), autism (aOR = 1.6, 95% CI [1.2, 1.9]), developmental delay (aOR = 2.0, 95% CI [1.7, 2.4]), intellectual disability (aOR = 1.8, 95% CI [1.2, 2.5]), learning disability (aOR = 1.9, 95% CI [1.7, 2.3]), speech disorder (aOR = 1.9, 95% CI [1.5, 2.3]), and Tourette syndrome (aOR = 3.2, 95% CI [1.7, 6.2]). All individual disorders showed significantly increased odds of having chronic pain than their counterparts without NDDs (*P* < 0.001 for all).

### 3.4. Associations between neurodevelopmental disorders and chronic pain by biological sex

We observed a main effect of sex with females having a higher probability of chronic pain than males, regardless of NDD status. In adjusted models, females with NDDs had 83% higher odds of chronic pain than males with NDDs (aOR = 1.8, 95% CI [1.6, 2.1], *P* < 0.0001). We did not find a statistically significant interaction between NDD status and biological sex on the odds of having chronic pain (aOR = 1.0, *P* = 0.79). The parallel slopes for females and males in Figure 2 visually represent this lack of significant interaction, suggesting that the magnitude of associations between NDDs and chronic pain is not moderated by sex. Consistent with this pattern, no individual NDD demonstrated a significant sex interaction in adjusted models (ADHD *P* = 0.44; autism *P* = 0.68; developmental delay *P* = 0.90; intellectual disability *P* = 0.70; learning disability *P* = 0.62; speech disorder *P* = 0.22; Tourette syndrome *P* = 0.23).

## 4. Discussion

In a national sample of U.S. children, the presence of neurodevelopmental disorders was consistently associated with elevated odds of chronic pain. This pattern held across every NDD diagnosis examined. We found no evidence that the association differed by sex.

Prior literature has demonstrated a high comorbidity of chronic pain and NDDs but is limited to clinical chronic pain samples or evaluation of single NDDs.^58^ Indeed, much of this larger literature cites heightened pain in ADHD and ASD populations.^36^ Our findings add to this by demonstrating elevated chronic pain in youth with other NDDs, with particularly elevated rates in youth with Tourette syndrome. There is evidence that repetitive motor tics, a characteristic of Tourette syndrome, can produce musculoskeletal strain and headaches,^33^ and that suppression of these tics, to blend in with neurotypical peers, may generate muscle tension and worsen their pain.^50^ However, recent genetic studies suggest that Tourette syndrome, ADHD, and autism share overlapping genetic risk factors, pointing to common neurodevelopmental pathways that may warrant further study in relation to pain outcomes.^60^

Research has demonstrated a substantial overlap between ADHD and chronic pain conditions. In tertiary pediatric pain clinics, 19.9% of pain patients exhibited clinically significant ADHD symptoms.^36^ Children with ADHD are found to have higher rates of headache and chronic stomach pain.^26,32^ Adults with ADHD report significantly more pain than their non-ADHD counterparts, with 80% reporting widespread pain,^30,34,48,49^ and, conversely, as many as 45% of fibromyalgia patients having ADHD.^16,45,56^ Across pediatric and adult literature, population studies and scoping reviews consistently report both elevated ADHD rates in chronic pain cohorts and greater chronic pain prevalence among individuals with ADHD.^6,36,54,59^ Mechanistically, those with ADHD may have impaired temperature sensation and sharp dull discrimination, as well as lower pain thresholds and increased sensitivity.^6,47,53^ Individuals with ADHD are also believed to have more muscular dysregulation and possibly neuroinflammation, which correlate with pain severity.^37,54^ Genetic studies have revealed positive genetic associations between ADHD and certain types of chronic pain: both may share genetic architecture related to dopaminergic and noradrenergic pathways affecting both attention regulation and descending pain modulation.^14,23,27,37^

As with ADHD, a large portion of patients in tertiary pediatric pain clinics also present with autism traits (13.7%).^36^ Whitney and Shapiro^58^ found that children with ASD had over twice the odds of experiencing pain compared with those without, and children with both ASD and additional developmental comorbidities had even higher odds. However, both hypersensitivity and hyposensitivity to painful stimuli have been reported in ASD populations.^1,39^ Physiological explanations for hyposensitivity have been postulated^1^; however, this is more likely attributed to atypical pain responses leading to poor external recognition, as individuals with ASD have been shown to have both greater physiological responses to pain stimuli, and hypersensitive pain threshold and pain intensity ratings.^4,11,18,41,51^ Altered sensory processing may contribute to these phenomena, as sensory abnormalities are a diagnostic criterion for ASD.^2,7^ Neurological explanations as well as genetic links to irritable bowel syndrome and multisite pain have also been proffered.^7,13,35^

Chronic pain is common across many NDDs but is frequently underrecognized due to communication barriers.^5^ Intellectual disability is one such example^57^; previous studies have shown that some individuals with intellectual disabilities demonstrate heightened reactivity to noxious input and greater pain-evoked responses, which can predispose them to developing central sensitization over time.^5^ Alternatively, when examining learning disabilities, poor childhood reading ability is associated with greater pain of various types, including headache, abdominal pain, eye pain, back pain and rheumatism, although this relationship may be influenced by socioeconomic status.^10^ Finally, motor proficiency delays have high co-occurrence with chronic pain-related disability, and, across NDD disorders, joint hypermobility and autonomic dysfunction are believed to contribute to pain.^15,19,54^ Moreover, across these disorders, common themes such as increased sleep disturbance and heightened anxiety can maintain and amplify pain over time,^3^ illustrating how various biopsychosocial factors intersect to contribute to chronic pain. These factors often operate in a bidirectional manner, where one challenge exacerbates the other. For instance, chronic sleep fragmentation can heighten sensory sensitivities, while persistent anxiety may amplify the emotional impact of physical pain. This self-perpetuating cycle not only sustains the pain experience but can also progressively diminish a child's capacity for social and academic engagement.

As our data show, individuals with NDDs have significantly increased odds of having chronic pain. However, measurement discrepancies may help to explain underdiagnosis in these populations. Children with NDDs can express pain atypically and caregivers interpret those behaviors through their own lens,^22^ allowing for bias and underreporting of pain. One study of children with autism found that although they displayed strong facial pain reactions during pain stimulations, caregiver ratings did not align with those observed behaviors, emphasizing the risk of under recognition when relying solely on parental report.^41^ To reduce misclassification, routine pain assessment in neurodevelopmental care should blend child self-report when feasible with caregiver report and clinical observation. This can include integrating routine pain screenings such as the Non-Communicating Children's Pain Checklist, which has been proven to be reliable for measuring pain in children with cognitive impairments,^9^ or the Paediatric Pain Profile, a validated behavioral pain assessment scale developed specifically for children with severe neurological and communication impairments.^25^ Incorporating these assessment tools into large surveys may reveal even stronger associations than those detected with a single caregiver question.

Better measurement translates into better care when it is paired with targeted education. Although standardized pain assessment tools exist, they are not always used routinely, in part because clinicians may be unfamiliar with available tools or uncertain about how to interpret pain behaviors in children with communication differences. This uncertainty often leads clinicians to rely on parents and caregivers who may be best positioned to recognize deviations from a child's baseline behavior.^5^ Therefore, effective pain assessment requires shared communication between families and healthcare teams. Caregivers can benefit from concrete guidance on how pain may look different in NDDs and how to document changes from a child's baseline.^41^ Similarly, clinicians need targeted training in pain recognition and management for neurodivergent populations. In prior work, it was found that only a small fraction of nurses working with individuals with intellectual disabilities report receiving formal education in pain management for youth with NDDs.^17^ Our finding that chronic pain is elevated across multiple NDDs suggests value in explicitly incorporating NDD-sensitive pain recognition into pediatric and behavioral health training.

Chronic pain is more prevalent in girls in the general pediatric population,^12^ and as expected, our results showed that females had higher odds of pain overall. However, our analysis did not identify any moderating effect of biological sex on the association between NDDs and chronic pain. Some prior work has suggested that sex differences in pain-like symptoms may persist in certain NDD subgroups: in a large ADHD sample, boys and girls were similar on most clinical variables, but girls rated higher on somatic complaints, a broad symptom category that can include pain.^21^ Broader somatic complaints domain may capture sex-linked differences in symptom perception or reporting that are not as apparent when using a stricter chronic pain definition that we used in our study. In addition, the inclusion of a broad pediatric age range may have attenuated potential sex differences, as disparities in pain are most pronounced during adolescence and less evident in younger children.^42^ Despite there being a larger body of literature examining NDDs within pediatric chronic pain populations, to our knowledge, this is the first study to examine prevalence rates of chronic pain in DSM-5 NDDs using national survey data. Use of a large, nationally representative data set and a consistent DSM-5–aligned exposure across multiple neurodevelopmental diagnoses improve comparability within clinical literature.

Our findings should be interpreted in light of several limitations. First, chronic pain was measured with a single caregiver-reported item, which may produce misclassification, particularly for children with limited verbal skills. In populations with communication differences, caregiver reports and child reports of pain often diverge.^22^ Second, the NSCH chronic pain item bundled “frequent or chronic” pain and explicitly referenced headaches and other back or body pain. The lack of detail did not give us information on the duration, frequency, severity, or location of the pain. This may preferentially capture more severe or functionally significant pain and may therefore underestimate the broader burden of pediatric chronic pain. In addition, children younger than 5 years were included in the analytic sample. This age group is often underrepresented in chronic pain prevalence research because of developmental limitation in pain self-report and the lack of validated proxy-based definitions of chronic pain for young children, so their inclusion may have attenuated overall prevalence estimates. Third, the demographic composition of our sample was predominantly White, English-speaking, privately insured, and of medium to high income. Although we controlled for these variables in our multivariable analyses, the potential for sample selection bias cannot be fully excluded. Therefore, our findings may have limited generalizability to children from more diverse racial, ethnic, and socioeconomic backgrounds. Finally, the cross-sectional design of the data limits causal interpretation of associations between NDDs and chronic pain. Future longitudinal studies are needed to delineate the temporal sequencing of these conditions.

In conclusion, children with neurodevelopmental disorders within this national sample had significantly higher odds of chronic pain compared with their peers without NDDs. This elevated odds was consistent across all NDDs examined and did not differ by sex. Our findings emphasize the need for better understanding of the mechanisms underlying chronic pain in youth with neurodevelopmental disorders—an area which has gained recent traction, but which remains understudied—to facilitate tailored pain assessments and management strategies in pediatric developmental care.

## Disclosures

The authors have no conflict of interest to declare.
